# Case Report: Reversible neurological involvement associated with Chlamydia psittaci infection: clinical, cerebrospinal fluid and neuroimaging features of three fully documented cases

**DOI:** 10.3389/fmed.2026.1932046

**Published:** 2026-08-28

**Authors:** Hao Zhang, Jia Zhou, Yong Chen, Xiuyan Wang, Xiaoping Luo, Guilan Yang, Zhenzhen Gui, Shuni Wang, Yanan Zhang, Juan Chen

**Affiliations:** 1 Ningxia Key Laboratory of Clinical and Pathogenic Microorganisms, Institute of Medical Science, General Hospital of Ningxia Medical University, Yinchuan, China; 2 Department of Respiratory and Critical Care Medicine, General Hospital of Ningxia Medical University, Yinchuan, China

**Keywords:** cerebrospinal fluid, Chlamydia psittaci, CLOCCs, metagenomic next-generation sequencing, neurological involvement, opening pressure, sepsis-associated encephalopathy

## Abstract

**Background:**

Chlamydia psittaci infection presents mainly as severe pneumonia, but a subset of patients develop neurological symptoms. Previous reports are largely single cases and frequently lack cerebrospinal fluid (CSF) data, opening pressure and follow-up imaging, which makes the mechanism of neurological involvement difficult to determine.

**Methods:**

We retrospectively analysed three patients with C. psittaci infection confirmed by metagenomic next-generation sequencing (mNGS), all of whom developed neurological manifestations and had complete clinical, CSF, neuroimaging and follow-up data. Exposure history, neurological phenotype, laboratory and CSF parameters, opening pressure, treatment and outcome were collected. Potential explanations for neurological involvement were interpreted in the context of the clinical findings and published literature.

**Results:**

All three patients (two men and one woman; aged 50–72 years) reported pigeon or poultry exposure. Two presented with encephalopathy/delirium and one with headache. Opening pressures were 160, 230 and 90 mmH₂O; none exceeded 250 mmH₂O. CSF white-cell counts were 0–2/mm³, with mild protein elevation in one patient, and the CSF pathogen tests performed were negative. This mismatch between neurological manifestations and routine CSF findings met the study's operational description of CSF–clinical dissociation. Two patients had reversible splenial lesions consistent with cytotoxic lesions of the corpus callosum (CLOCCs). Hypoalbuminemia was present in all three patients, whereas hyponatremia and coagulation abnormalities occurred in selected cases. Neurological symptoms resolved within 4–7 days after pathogen-directed therapy, and all patients recovered completely during follow-up.

**Conclusions:**

In these three fully documented cases, C. psittaci-associated neurological involvement was short-lived and reversible. The clinical–CSF mismatch was consistent with the hypothesis that unmeasured indirect processes may have contributed, but it did not establish a mechanism or exclude low-burden direct CNS infection. Reversible splenial lesions consistent with CLOCCs were a notable imaging finding. Co-detected organisms and systemic complications limited pathogen-specific causal attribution. Clinical improvement was temporally associated with pathogen-directed therapy.

## Background

Chlamydia psittaci is an obligate intracellular zoonotic pathogen; humans are usually infected by inhaling aerosols from birds or poultry (1). Its clinical presentation is non-specific and may progress from influenza-like illness to severe community-acquired pneumonia (CAP) and multiorgan dysfunction (2, 3). The increasing use of metagenomic next-generation sequencing (mNGS) has improved detection and revealed previously under-recognised cases (4, 5). Beyond the respiratory system, C. psittaci infection can cause headache, delirium, impaired consciousness, meningoencephalitis and Guillain–Barré syndrome (6–9). A few reports have detected pathogen sequences in CSF, suggesting possible direct central nervous system (CNS) invasion in selected patients (7, 8). In other patients, neurological manifestations accompanying severe pneumonia may be compatible with systemic inflammation, metabolic disturbance, coagulopathy or other reversible processes. Neurological symptoms alone are insufficient to establish direct CNS infection; CSF findings, opening pressure, neuroimaging and the clinical course should be interpreted together.

A major limitation of previous studies has been incomplete data—missing lumbar puncture, CSF biochemistry or follow-up imaging—which weakens mechanistic inference. The present study describes three patients with complete CSF, opening-pressure, neuroimaging and follow-up data. By integrating these observations, we aimed to characterise the clinical spectrum and to assess whether the available findings were more consistent with direct CNS infection or with indirect, reversible neurological dysfunction. Given the descriptive design and small sample, the analysis was intended to provide supportive, hypothesis-generating evidence rather than mechanistic proof.

## Patients and methods

### Patients

This was a single-centre retrospective case analysis. Inclusion criteria were: (i) C. psittaci infection confirmed by mNGS of bronchoalveolar lavage fluid (BALF) or sputum; (ii) clear neurological symptoms or signs during the course; and (iii) complete CSF, opening pressure, neuroimaging and follow-up data. All three patients were hospitalised at the General Hospital of Ningxia Medical University between 2021 and 2025 and completed at least one month of follow-up.

### Data collection

We collected demographics, comorbidities, pigeon/poultry exposure, time from onset to admission, presenting symptoms, peak temperature, neurological manifestations, admission Glasgow Coma Scale (GCS), meningeal signs, inflammatory and metabolic indices, CSF routine and biochemistry, opening pressure, CSF pathogen testing, head and chest imaging, specimen type and sequence reads, antimicrobial regimens, time to neurological resolution, length of stay and follow-up outcome.

### Definitions

Neurological phenotype was classified as encephalopathy/delirium (type I), headache-predominant (type II) or cerebellar/ataxic. Opening pressure was recorded in mmH₂O as an indirect index of intracranial pressure, with 60–250 mmH₂O used as the interpretive reference range. CLOCCs were defined as splenial restricted diffusion (DWI hyperintensity with reduced ADC) in a non-vascular distribution, usually showing reversibility after control of the underlying disorder (10, 11). The term “CSF–clinical dissociation” is not used here as an established neurological diagnosis; it is an operational descriptive definition proposed for this study, denoting marked neurological symptoms accompanied by negative CSF pathogen testing, no definite intracranial hypertension, and routine CSF changes that are mild or disproportionate to clinical severity. MRI examinations were jointly reviewed by a specialist radiologist and a neurologist. The neurologist was aware of the patients’ clinical information at the time of image review; therefore, the imaging assessment was not blinded. The final imaging interpretation was reached by consensus between the radiologist and the neurologist after discussion of the MRI findings in the clinical context. Descriptive statistics were used.

## Results

### Baseline data and neurological phenotype

**Figure 1:**
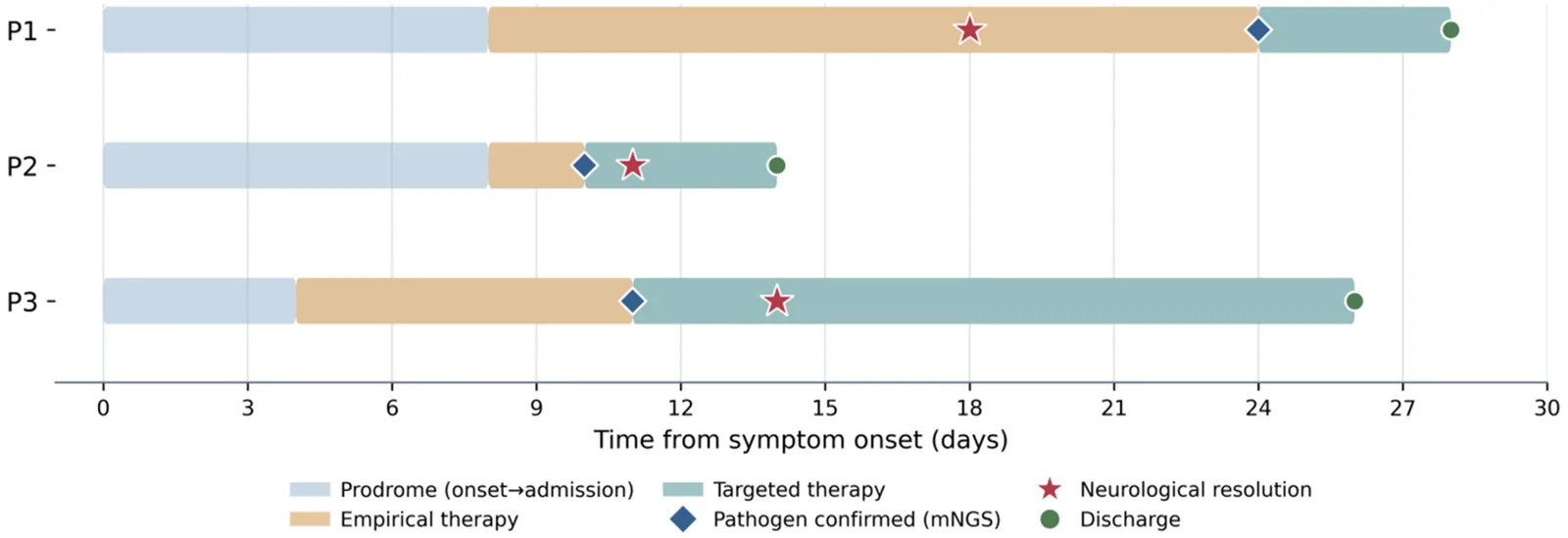
Clinical course timeline of the three patients. The illness-day timelines were reconstructed from charted events; treatment and neurological-resolution markers are approximate and should not be used to calculate exact treatment-to-resolution intervals.

The three patients (two men, one woman; aged 72, 50 and 53 years) all had clear pigeon or poultry exposure (P1 kept pigeons and a parrot; P2 and P3 had pigeon contact). Time from onset to admission was 4–8 days. P1 and P2 had the encephalopathy/delirium phenotype with abnormal behaviour, visual hallucinations, incoherent speech and confusion; P3 was headache-predominant with high fever and severe headache. Meningeal signs were negative in all, and admission GCS was 10–15 (Figure 1 and Table 1).

**Table 1:** Baseline data, exposure history and neurological phenotype.

ID	Age/sex	Comorbidity	Exposure	Onset to adm. (d)	Peak T (°C)	Phenotype	GCS
P1	72/M	Prior SAH	Pigeons + parrot	8	39.0	I (enceph./delirium)	14
P2	50/F	None	Pigeons	8	38.6	I (enceph./delirium)	10
P3	53/M	Type 2 diabetes	Pigeons	4	40.6	II (headache)	15

SAH, subarachnoid haemorrhage; GCS, glasgow coma scale. P1–P3 are de-identified codes.

**Figure 2:**
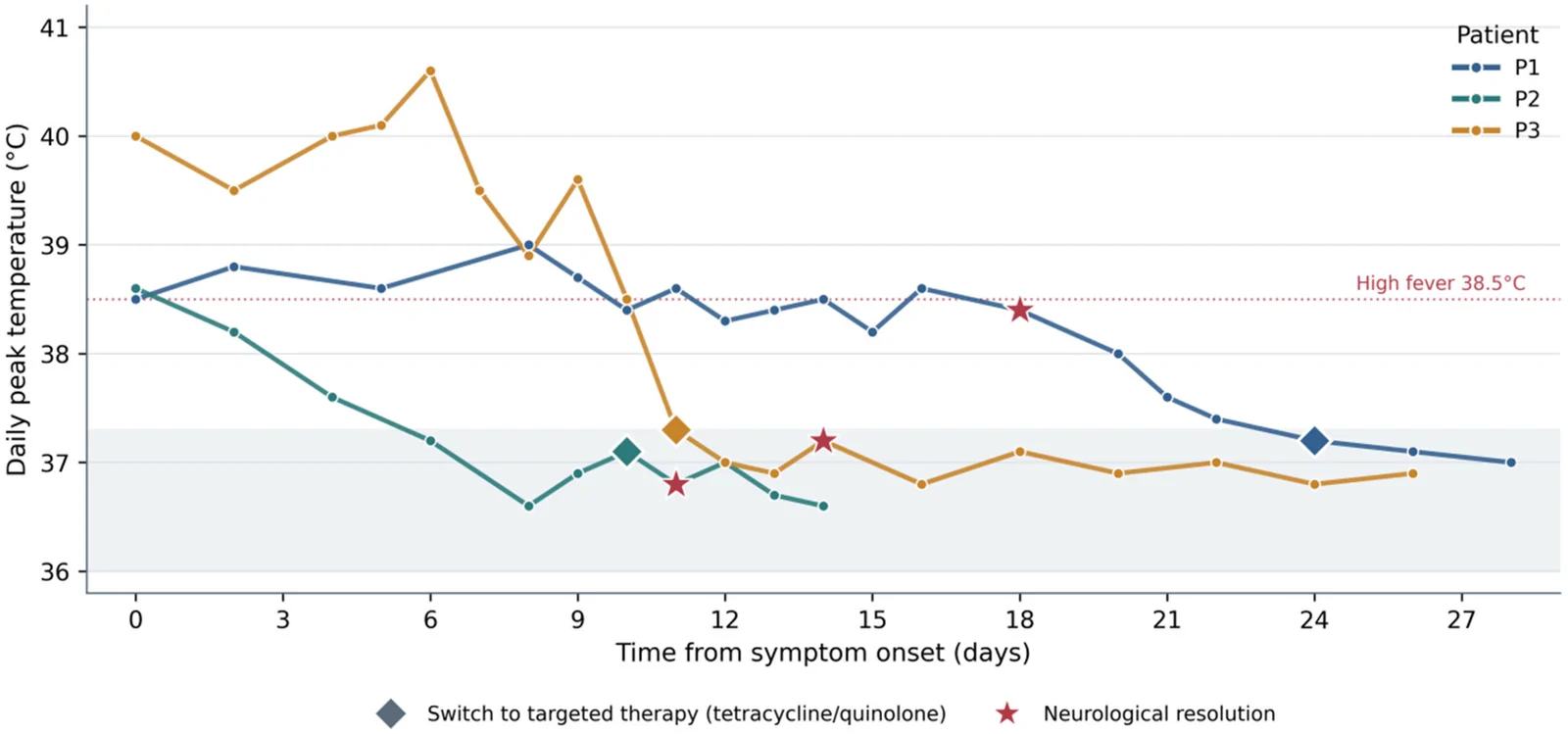
Body temperature and response to antimicrobial therapy. Temperature points and event markers were reconstructed from the clinical record; marker positions are approximate and do not establish causality.

**Figure 3:**
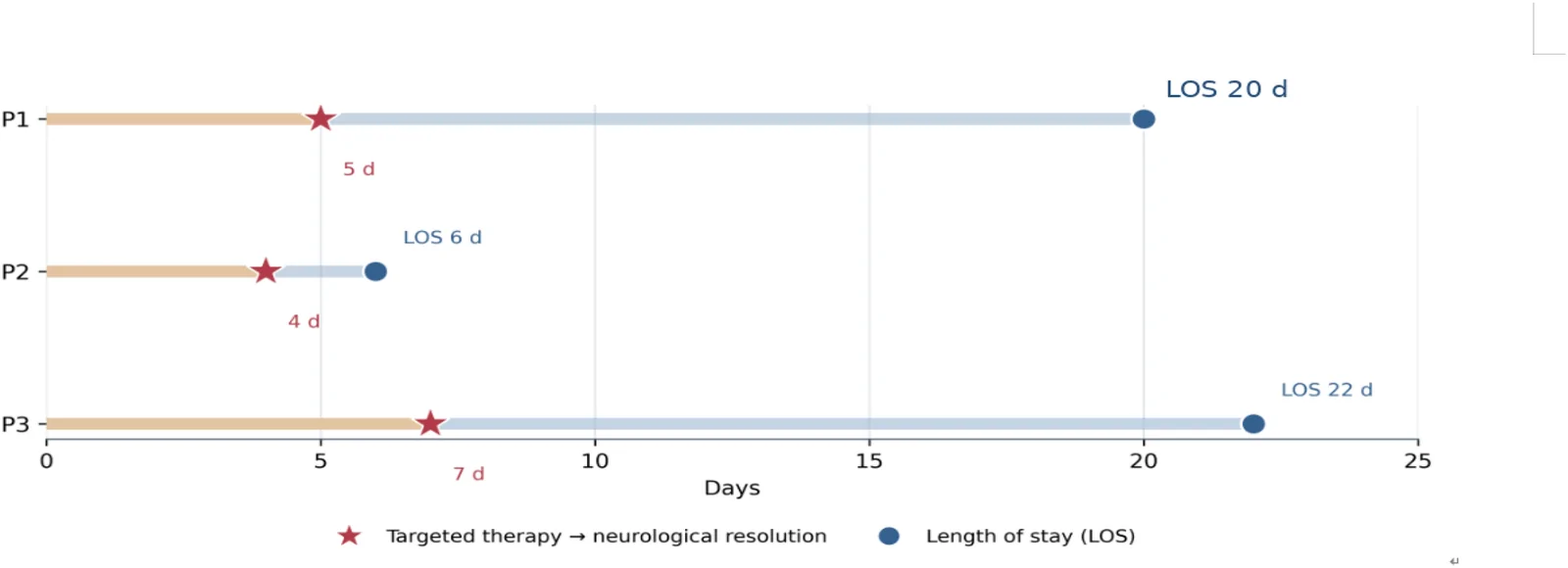
Time to neurological resolution and length of stay (all three fully recovered).

All three patients had persistent or relapsing fever during hospitalisation. P3 reached a peak temperature of 40.6 °C and became afebrile within 2–3 days after omadacycline plus doxycycline was started. Figure 2 is a reconstruction of charted temperature peaks and approximate event timing; it illustrates temporal context but should not be used to calculate exact treatment-to-resolution intervals or infer treatment causality. The chart-derived treatment-to-resolution intervals used for descriptive reporting are presented separately in Figure 3 and Table 2.

**Table 2:** Pathogen, treatment and outcome.

ID	Specimen	Reads	Co-detected organism	Targeted regimen	Resol. (d)	LOS (d)	Outcome
P1	BALF	1,023	None	Moxifloxacin	5	20	Survived/full recovery
P2	BALF	425	EBV	Moxifloxacin + doxycycline	4	6	Survived/full recovery
P3	Sputum	80,007	S. pneumoniae	Omadacycline + doxy-cycline + steroid	7	22	Survived/full recovery

Resol, time from targeted therapy to neurological resolution; LOS, length of stay; EBV, Epstein–Barr virus.

### Inflammatory, metabolic and coagulation indices

Inflammatory abnormalities varied among the three patients. Neutrophil proportions ranged from 65.1% to 91.7%. CRP was elevated in P1 and P2 but was not measured in P3; PCT was highest in P3 (2.90 ng/mL). All three had hypoalbuminemia (nadir 21.9–30.0 g/L), and P1 and P3 had hyponatremia (nadir 125 and 130.7 mmol/L). D-dimer was elevated to varying degrees and peaked at 9.78 mg/L FEU in P3; P3 also had a fibrinogen peak of 9.36 g/L. These results document inflammatory, metabolic and coagulation abnormalities but do not establish their causal contribution to the neurological manifestations (Table 3).

**Table 3:** Main inflammatory, metabolic and coagulation indices.

ID	WBC (×10⁹/L)	NEUT %	CRP (mg/L)	PCT (ng/mL)	Albumin (g/L)	Sodium (mmol/L)	D-dimer (mg/L)
P1	7.72	76.4	188.4	0.33	29.8	125	2.01
P2	6.30	65.1	55.9	0.12	30.0	138	1.49
P3	8.03	91.7	—	2.90	21.9	130.7	9.78

“—”, not measured. Albumin and sodium are the lowest in-hospital values.

### CSF and opening pressure: the CSF–clinical dissociation

**Figure 4:**
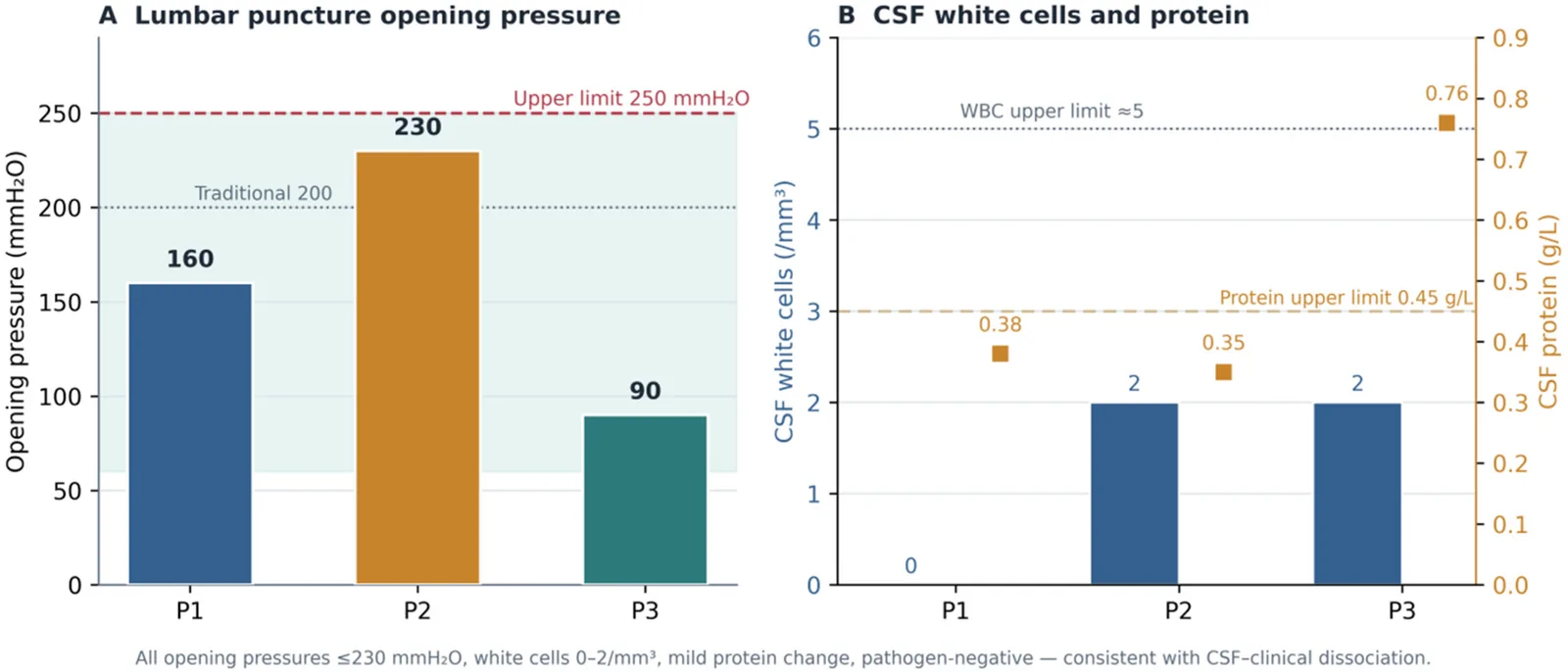
CSF white cells, protein and lumbar-puncture opening pressure in the three patients. Panel **(A)**: Lumbar puncture opening pressure; Panel **(B)**: CSF white cells and protein. “CSF–clinical dissociation” is used only as the operational descriptive term proposed in this study. “Pathogen-negative” refers only to the CSF pathogen tests that were performed and does not exclude low-burden CNS infection.

All three underwent lumbar puncture with pressure measurement; opening pressures were 160 (P1), 230 (P2) and 90 (P3) mmH₂O. Only P2 exceeded the traditional 200 mmH₂O threshold, and all values remained below 250 mmH₂O (12), providing no evidence of definite intracranial hypertension. CSF white-cell counts were 0–2/mm³, without pleocytosis; protein was 0.35–0.76 g/L. P3's mild protein elevation occurred in the context of poorly controlled diabetes and may have reflected non-specific blood–brain-barrier dysfunction, but its cause cannot be determined from these data. CSF glucose and chloride did not show a pattern typical of purulent or fungal meningitis, and the performed CSF pathogen tests and referred autoimmune-encephalitis antibody tests were negative. P2 had the lowest admission GCS (10) and the most severe delirium despite near-normal routine CSF findings and only mildly elevated opening pressure. Collectively, the three cases met the study's operational description of CSF–clinical dissociation; this pattern supports, but does not prove, an indirect process and cannot exclude low-burden direct CNS infection because CSF mNGS was not routinely performed (Figure 4 and Table 4).

**Table 4:** CSF findings, opening pressure and interpretation.

ID	OP (mmH₂O)	WBC (/mm³)	Protein (g/L)	Glucose (mmol/L)	Chloride (mmol/L)	Pathogen	Interpretation
P1	160	0	0.38	5.0	115	Negative	OP and routine essentially normal
P2	230	2	0.35	3.0	124	Negative	OP mildly high, <250
P3	90	2	0.76	4.2	122	Negative	Mild protein rise; rest normal

OP, opening pressure. The CSF pathogen tests performed and referred autoimmune-encephalitis antibody tests were negative. P3 had a mild, non-specific CSF protein elevation; its cause could not be determined from the available data.

### Neuroimaging

P1's brain MRI showed band-like splenial DWI hyperintensity with a right periventricular lacune and Fazekas grade 2 white-matter change; the non-vascular morphology and interval regression were consistent with an infection- or metabolic-associated CLOCC. P3's external MRI had been reported as an “acute-phase rounded infarct” in the splenium. Its midline rounded morphology, lack of conformity to a single vascular territory and complete resorption on follow-up favoured a CLOCC over acute ischaemic infarction, although retrospective classification without a blinded independent neuroradiology review warrants caution. P2's contrast brain MRI showed no parenchymal abnormality, demonstrating that neurological symptoms in this small series were not invariably accompanied by a splenial lesion (Table 5).

**Table 5:** Neurological manifestations and head imaging.

ID	Neurological features	Conscious-ness	Meningeal signs	Main imaging finding	Splenial involve	Lesion resorbed
P1	Abnormal behaviour, hallucinations, incoherence	Alert	Negative	Splenial hyperintensity, Fazekas grade 2	Yes	Yes
P2	Abnormal behaviour, confusion, agitation	Confused	Negative	No parenchymal abnormality	No	Not applicable
P3	High fever, severe headache	Alert	Negative	Midline splenial lesion; outside report favoured infarct	Yes	Yes

**Figure 5:**
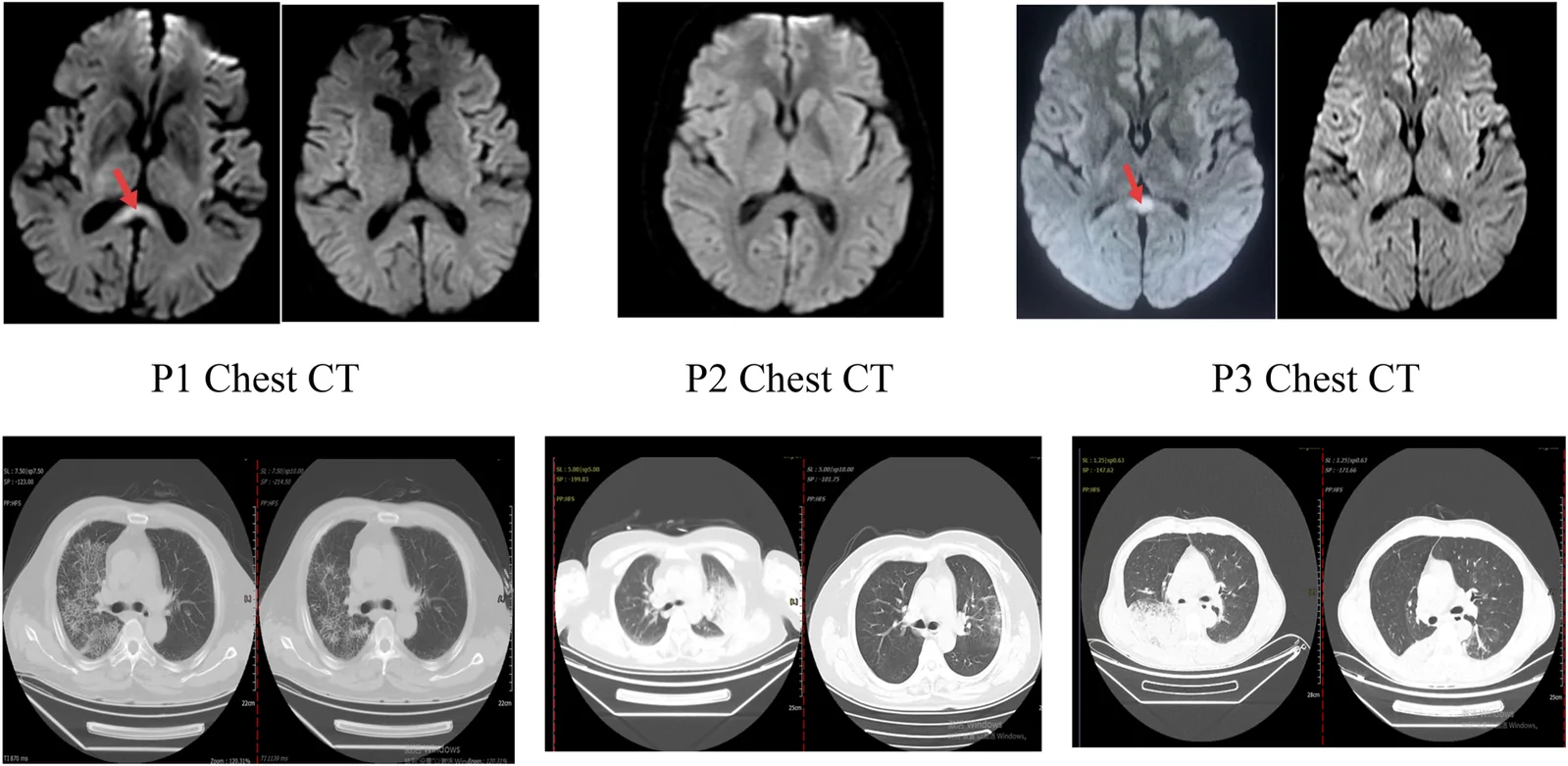
Available brain MRI and paired baseline/follow-up chest CT images. Brain MRI is shown at baseline and follow-up for P1 and P3 and at baseline only for P2. Arrows indicate the splenial DWI abnormalities in P1 and P3. All displayed images are de-identified.

P1 and P3 had splenial lesions that regressed on follow-up and were consistent with CLOCCs; arrows indicate the relevant DWI abnormalities in Figure 5. Classification remains interpretive and would be strengthened by blinded review of the complete original DWI/ADC series.

Chest CT in all three showed patchy/consolidative lesions consistent with CAP (P1 right upper lobe posterior segment; P2 left lung and right upper lobe; P3 right upper and lower lobes), with no pleural or pericardial effusion, and the pulmonary lesions resolved at one-month follow-up. Figure 5 shows baseline and follow-up brain MRI for P1 and P3, baseline brain MRI only for P2, and paired baseline/follow-up chest CT images for all three patients.

### Pathogen, treatment and reversible outcome

Confirmatory specimens were BALF for P1 and P2 and sputum for P3; C. psittaci mNGS read counts were 1023, 425 and 80,007, respectively. P2 also yielded Epstein–Barr virus and P3 Streptococcus pneumoniae; pathogenicity or mixed infection was judged from specimen type, read count, and clinical and imaging context. Empirical therapy included *β*-lactam agents; after confirmation, treatment included a quinolone and/or tetracycline (P1 moxifloxacin; P2 moxifloxacin plus doxycycline; P3 doxycycline plus omadacycline, with short-course methylprednisolone 40 mg/day). The chart-derived intervals from targeted therapy to neurological resolution were 5, 4 and 7 days for P1, P2 and P3, respectively, as summarised in Figure 3 and Table 2; because Figures 1, 2 are reconstructed timelines with approximate event markers, exact intervals should be taken from Table 2 rather than calculated from marker positions. Length of stay was 6–22 days. All three patients survived and were discharged with GCS 15 and neurological function returned to baseline. Follow-up imaging showed regression/resolution of the splenial lesions in P1 and P3 and resolution of the pulmonary lesions in all three patients.

## Discussion

The principal contribution of this series is the integration of CSF and opening-pressure data, longitudinal neuroimaging, and treatment–response timing within the same patients. The combined pattern was more consistent with short-lived, infection-associated neurological dysfunction than with typical pyogenic meningitis or destructive encephalitis. This remains an interpretation rather than proof of mechanism, particularly because the series contains only three patients, CSF mNGS was not routinely performed, and two patients had co-detected pathogens.

### From “neurological symptoms present” to evidence stratification: the value of CSF and opening pressure

Published reports include occasional detection of C. psittaci sequences in CSF, supporting possible direct invasion in some patients (7, 8). In contrast, the present cases showed no pleocytosis, no definite intracranial hypertension and negative results on the CSF pathogen tests that were performed. We use “CSF–clinical dissociation” only as an operational description of this mismatch, not as a recognised neurological entity or a validated mechanistic marker. The pattern is compatible with indirect neurological dysfunction, but negative routine CSF studies do not exclude low-burden infection, especially because CSF mNGS was not routinely performed. Accordingly, these observations should be used to stratify diagnostic probability rather than to rule out direct CNS invasion.

### Potential indirect contributors suggested by case-level clinical observations

**Figure 6:**
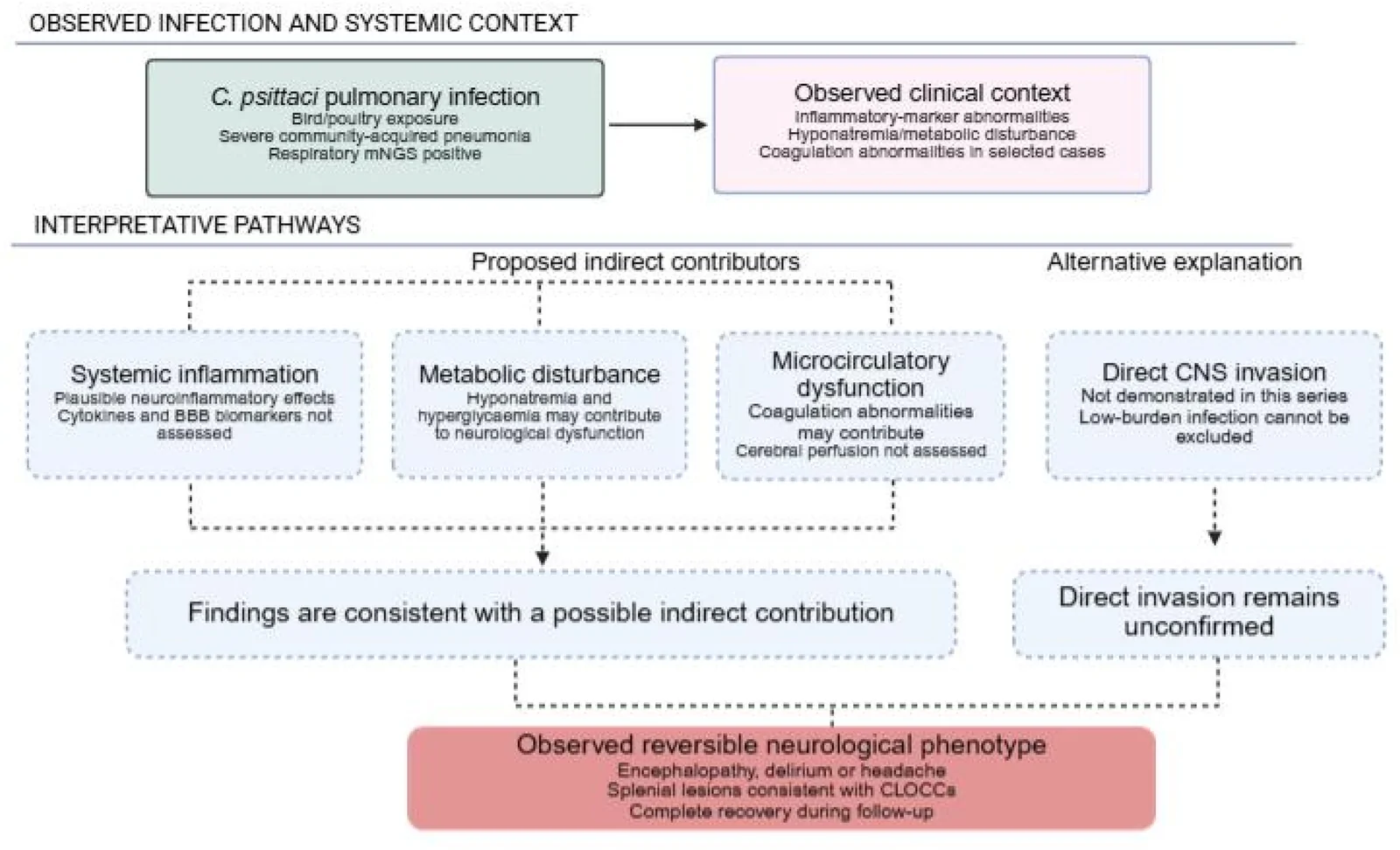
Hypothesis-generating model of potential pathways contributing to Chlamydia psittaci-associated reversible neurological dysfunction. The observed clinical abnormalities were consistent with possible indirect contributions from systemic inflammation, metabolic disturbance and microcirculatory dysfunction, although these mechanisms were not directly investigated. Direct CNS invasion was not demonstrated but cannot be excluded. Dashed arrows indicate hypothesized or unconfirmed associations rather than established causal pathways. BBB, blood–brain barrier; CLOCCs, cytotoxic lesions of the corpus callosum; CNS, central nervous system; mNGS, metagenomic next-generation sequencing.

Several observed abnormalities provide plausible, but untested, indirect pathways. Hyponatremia in P1 and P3 could have contributed to delirium or splenial cytotoxic oedema; hyperglycaemia in P3 may have added metabolic stress; and raised D-dimer/fibrinogen in P3 may have reflected coagulation and microcirculatory disturbance. Anaemia, hypoalbuminemia and systemic inflammation could also have reduced physiological reserve. However, this study did not measure cytokines, blood–brain-barrier biomarkers, cerebral perfusion or CSF inflammatory mediators. These pathways are therefore presented as hypotheses consistent with bedside data, not as established mechanisms or case-specific causal assignments (Figure 6).

Sepsis-associated encephalopathy (SAE) is diffuse brain dysfunction occurring in sepsis without evidence of direct CNS infection or another sufficient structural explanation; proposed mechanisms include neuroinflammation, blood–brain–barrier dysfunction, microcirculatory impairment, cerebral hypoxia and metabolic disturbance (9, 13). The neurological phenotype in this series shared some features described in SAE, particularly the association with severe systemic infection and metabolic abnormalities. However, SAE was not prospectively assessed or formally diagnosed, and hypoxia, cerebral perfusion, cytokines and blood–brain–barrier biomarkers were not systematically measured. The temporal association between pathogen-directed therapy and neurological recovery raises the possibility of treatment benefit but cannot establish causality or the underlying pathway. SAE remains a diagnosis of exclusion; appropriate evaluation should continue to exclude meningoencephalitis, cerebrovascular events, non-convulsive status epilepticus and metabolic or drug-related encephalopathy.

### Interpreting possible CLOCCs: the supportive but non-specific role of follow-up reversibility

CLOCCs are cytotoxic splenial lesions associated with diverse triggers, including infection, electrolyte or glucose disturbance, seizures and trauma; their characteristic non-vascular diffusion restriction and frequent reversibility help distinguish them from infarction (10, 11). In P1 and P3, lesion morphology and interval regression favoured CLOCCs. For P3, however, the outside-hospital interpretation of infarction, the hypercoagulable state and the absence of blinded central image review prevent definitive classification. We therefore describe both lesions as “consistent with CLOCCs.” Reversibility supports this interpretation but is not pathognomonic, and a minority of CLOCCs may leave residual abnormalities (14).

### Diagnostic and therapeutic implications: temporal clinical associations

For severe CAP with neurological symptoms, pigeon/poultry exposure should be sought and early aetiological testing considered (4, 5, 15). Fever and neurological manifestations improved during hospitalisation. Although Table 2 and Figure 3 summarise chart-derived intervals after pathogen-directed therapy, the reconstructed event markers in Figures 1,2 are approximate; these temporal observations cannot establish treatment causality. P2 had EBV detected and P3 had S. pneumoniae co-detected, so either co-infection, the combined inflammatory burden, or other unmeasured factors may have contributed to neurological manifestations. A causal relationship between C. psittaci and neurological dysfunction therefore cannot be established definitively in these two patients. mNGS findings must be interpreted with specimen type, read abundance, host context and conventional microbiology rather than assuming that every co-detected organism is causal. Diagnostic assessment should integrate CSF, opening pressure, MRI and metabolic/hypoxic factors while continuing to exclude alternative neurological disorders.

### Novelty and limitations

The uncommon strength of this series is the availability of CSF, opening-pressure, longitudinal imaging and outcome data in the same patients. These observations provide supportive evidence for a reversible neurological phenotype and generate the hypothesis that indirect processes may be important; they do not establish a new mechanistic entity. Major limitations are the sample size of three, retrospective selection of only fully documented cases, absence of routine CSF mNGS, lack of cytokine, blood–brain-barrier and cerebral-perfusion measurements, and lack of blinded independent neuroradiology review. Co-detection of EBV in P2 and S. pneumoniae in P3 further limits pathogen-specific causal attribution. Follow-up was limited to one month, and the temperature curves were reconstructed from recorded peaks and clinical events rather than continuous measurements. Future multicentre prospective studies should incorporate standardised neurological phenotyping, paired respiratory and CSF pathogen testing, biomarker assessment, blinded imaging review and longer cognitive follow-up.

## Conclusions

In this three-patient series, neurological involvement associated with C. psittaci infection was short-lived and reversible. The mismatch between clinical manifestations and routine CSF findings was compatible with the operational description of CSF–clinical dissociation and supported the hypothesis that indirect processes may have contributed; it did not exclude direct CNS infection. Splenial lesions in P1 and P3 were consistent with CLOCCs rather than definitively diagnostic. Clinical improvement followed targeted therapy, but the small retrospective design and co-infections preclude causal conclusions. Larger prospective studies with paired CSF testing, mechanistic biomarkers and blinded imaging review are required.

## Clinical takeaways

- In severe community-acquired pneumonia with neurological symptoms, actively ask about bird or poultry exposure and consider early testing for C. psittaci.
- Interpret neurological involvement by integrating CSF findings, opening pressure, MRI evolution and metabolic/hypoxic factors; negative routine CSF tests reduce but do not eliminate the possibility of direct CNS infection.
- A midline splenial diffusion-restricted lesion that regresses on follow-up is consistent with CLOCCs, but vascular and other mimics should be excluded.
- Co-detected pathogens and systemic complications may contribute to neurological manifestations; pathogen-specific causality should be stated cautiously.

## Data Availability

The raw data supporting the conclusions of this article will be made available by the authors, without undue reservation.
